# Practical guidance for the evaluation of disease progression and the decision to change treatment in patients with advanced gastric cancer receiving chemotherapy

**DOI:** 10.1007/s10147-020-01684-z

**Published:** 2020-04-29

**Authors:** Satoru Iwasa, Toshihiro Kudo, Daisuke Takahari, Hiroki Hara, Ken Kato, Taroh Satoh

**Affiliations:** 1 Gastrointestinal Medical Oncology Division, National Cancer Center Hospital, 5-1-1, Tsukiji, Chuo-ku, Tokyo, 104-0045 Japan; 2 Department of Medical Oncology, Osaka International Cancer Institute, Osaka, Japan; 3 Department of Gastroenterology, Cancer Institute Hospital of the Japanese Foundation for Cancer Research, Tokyo, Japan; 4 Department of Gastroenterology, Saitama Cancer Center, Saitama, Japan; 5 Frontier Science for Cancer and Chemotherapy, Osaka University Graduate School of Medicine, Osaka, Japan

**Keywords:** Gastric cancer, Prognostic factor, Tumor marker, RECIST

## Abstract

After failure of first-line chemotherapy with fluoropyrimidines and platinum compounds for advanced gastric cancer, second-line chemotherapy with ramucirumab plus paclitaxel, which elicits a durable response, and third-line or later chemotherapy with nivolumab have been shown to lead to a more favorable prognosis in advanced gastric cancer patients. As new and more effective drugs are now available, sequential chemotherapy would contribute to prolonged survival. From this point of view, the patient’s disease course should be frequently monitored in order to adapt treatment regimens. This review summarizes the points to note in regard to radiological assessment, and discusses the integration of prognostic factors, tumor markers, and clinical symptoms that need to be taken into account to change treatment at an appropriate timing.

## Introduction

Gastric cancer is the fifth most common malignancy and the third leading cause of cancer mortality worldwide [1]. Platinum compounds plus fluoropyrimidines are the most common first-line treatment for patients with unresectable advanced gastric cancer, with a median survival ranging from 8–10 months for patients with human epidermal growth factor receptor 2 (HER2)-negative disease [2–9].

In the second-line setting, taxanes (docetaxel or paclitaxel), or irinotecan are the validated therapeutic options for patients in good general condition [10–12]. More recently, two phase III trials have demonstrated that ramucirumab (an anti-vascular endothelial growth factor receptor 2 [VEGFR2] monoclonal antibody), as a single agent or in combination with paclitaxel, is associated with a survival benefit [13, 14].

Evidence showing an overall survival (OS) benefit of therapy in third- or later lines of chemotherapy in patients with advanced gastric cancer suggests that salvage therapy may indeed become the standard of care. The Asian ATTRACTION-02 phase III randomized trial comparing nivolumab (an anti-PD-1 antibody) to placebo in patients with unresectable advanced gastric cancer pretreated with two or more chemotherapy regimens has recently been published [15]. OS was significantly increased in the nivolumab group compared to the control group. A phase III trial conducted in China demonstrated a benefit with apatinib, a novel, orally administered VEGFR inhibitor, in the third-line setting [16]. Trifluridine/tipiracil (TAS-102) has also shown comparable efficacy as a third-line treatment [17].

In cross-comparisons of first-line treatment trials, treatment arms with a higher proportion of patients receiving subsequent treatments showed better OS compared to treatment arms in which less patients received subsequent lines of therapy (Table 1) [2–14, 18]. This has demonstrated the positive impact that subsequent treatments can have. This phenomenon was evident in the comparison between the JCOG9205 and JCOG9912 trials, in which similar progression-free survival (PFS) was achieved with the same first-line treatment consisting of 5-FU monotherapy, yet OS was longer in the JCOG9912 trial in which 80% of patients received later lines of treatment [19]. OS has improved with the development of drugs that are effective not only in first-line but also in later lines of treatment. In a systematic review of 25 phase III trials for gastric cancer, it was reported that a higher proportion of patients receiving subsequent chemotherapy correlates with a longer overall survival [20].

**Table 1:** Pivotal phase 3 trials in advanced gastric cancer

Trial	Arm	PFS (months)	OS (months)	RR (%)	Subsequent chemotherapy (%)
First-line chemotherapy					
JCOG9912	5-FU	2.9	10.8	9	83
	S-1	4.2	11.4	28	74
SPIRITS	S-1	4.0	11.0	31	75
	SP	6.0	13.0	54	74
ML17032	FP	5.0	9.3	32	24
	XP	5.6	10.5	46	24
AVAGAST	XP	5.3	10.1	37.4	45
G-SOX	SP	5.4	13.1	52.2	84.7
	SOX	5.5	14.1	55.7	84.3
JCOG1013	SP	6.5	15.3	56	79
	DCS	7.4	14.2	59	77
ToGA*	XP/FP	5.5	11.1	35	43
	XP/FP + Tmab	6.7	13.8	47	38
JACOB*	XP + Tmab	7.0	14.2	48.3	42
	XP + Tmab + pertuzumab	8.5	17.5	56.7	43
Second-line chemotherapy					
COUGAR-02	ASC	–	3.6	–	19
	DTX	–	5.2	7	8.3
WJOG4007	PTX	3.6	9.5	20.9	89.8
	IRI	2.3	8.4	13.6	72.1
REGARD	BSC	1.3	3.8	3	39.3
	RAM	2.1	5.2	3	31.5
RAINBOW	PTX	2.9	7.4	16	46
	PTX + RAM	4.4	9.6	28	48
ABSOLUTE	PTX	3.8	10.9	24	77
	nab-PTX (q1w)	5.3	11.1	33	70
	nab-PTX (q3w)	3.8	10.3	25	72
Third- or later-line chemotherapy					
ATTRACTION-2	BSC	1.45	4.14	0	44.2
	Nivolumab	1.61	5.26	11.2	47.0
TAGS	BSC	1.8	3.6	2	25
	FTD/TPI	2.0	5.7	4	26

PFS: progression-free survival; OS: overall survival; RR: response rate; 5-FU: fluorouracil; SP: S-1 plus cisplatin; FP: 5-FU plus cisplatin; XP: capecitabine plus cisplatin; SOX: S-1 plus oxaliplatin; DCS: docetaxel and cisplatin plus S-1; ASC: active symptom control; DTX: docetaxel; PTX: paclitaxel; IRI: irinotecan; BSC: best supportive care; RAM: ramucirumab; nab-PTX: nab-paclitaxel; Tmab: trastuzumab; FTD/TPI: trifluridine/tipiracil

*Patients with HER2-positive metastatic gastric or gastroesophageal junction cancers were included in these trials

A post hoc analysis of the Japanese subpopulation from the RAINBOW trial showed that patients with measurable disease who received second-line ramucirumab plus paclitaxel had a response rate of 41.2% and a disease control rate of 94.1% [21]. This is surprisingly comparable to the results achieved with first-line chemotherapy such as a fluoropyrimidine plus a platinum compound (regardless of HER2 status) [22].

Data from the Japanese subpopulation in Attraction 2 trial shows objective response rate of 14% and duration of response of 14.5 months that is better than the intention-to-treat population. Japanese patients who treated with prior ramucirumab therapy indicated higher objective response rate (22.2%) and better OS (hazard ratio of 0.57) [23]. These data suggest importance of treatment sequence and treatment choice.

In the light of such findings, it is no longer true that first-line therapy is the last line of treatment for advanced gastric cancer. Many patients can now expect similar efficacy and benefit not only from first-line but also from later lines of therapy.

**Fig. 1:**
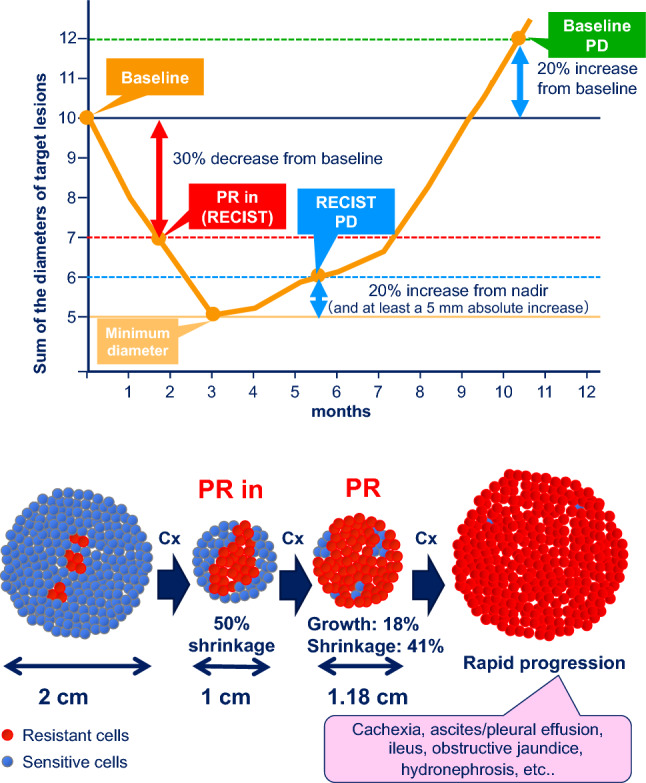
Tumor assessment and the relationship between sensitive and resistant cells. **a** Tumor response according to RECIST. **b** Important points of tumor assessment. PR: partial response, PD: progressive disease, PR in: initial partial response, Cx: chemotherapy

Figure 1a shows the conceptual diagram of tumor response according to the “Response evaluation criteria in solid tumors (RECIST)” guidelines and baseline progressive disease (PD) [24]. In the example shown, the therapy was started at baseline and a favorable effect was observed initially; however PD occurred gradually or diminished efficacy was observed. The initial 30% tumor regression is defined as “partial response (PR)” in RECIST. The presence of a subsequent 20% increase from the minimum size and an increase by at least 5 mm in absolute value is defined as “PD” in RECIST. The return to baseline is defined as “baseline PD”. Medical oncologists are taught to make the most of RECIST when evaluating the effect of therapy and when considering the timing of therapy change. However, excessive adherence to RECIST may result in missing the appropriate timing for switching to second- or third-line therapy in the treatment of advanced gastric cancer.

When the patient starts treatment, there are few resistant cells, and a PR state is achieved through chemotherapy. With continued PR, the tumor initially regresses (for example from 2 cm to 1 cm) before an increase (for example to 1.18 cm) is recorded at the next evaluation of efficacy, and the PR state is maintained. Since the tumor is smaller, the patient is categorized as having stable disease (SD). However, it is critical to know if this smaller tumor is now comprised mainly of resistant cells that emerged as a direct result of treatment (Fig. 1b). Otherwise, a sudden progression of the cancer may be observed prior to or at the next scanning occasion, which is more frequently observed in gastric cancer than in other cancer types.

Although there is mounting evidence of the importance of second-line and salvage chemotherapy, there is little published guidance on how to utilize subsequent treatment at the appropriate timing. In this review, we discuss ways to increase the proportion of patients getting the most benefit from subsequent lines of treatment. We focus on the timing of radiographic assessment, and the evaluation of tumor markers, prognostic factors and clinical symptoms, all of which may be useful in determining disease progression and clinical outcomes.

## Prognostic factors

There are several prognostic factors that might be useful for monitoring patients during chemotherapy.

Two major reports analyzing prognostic factors during first-line treatment have been published. The first identifies the Royal Marsden Hospital prognostic Index (RMH-Index); performance status (PS) ≥ 2, liver metastases, peritoneal metastases, and alkaline phosphatase (ALP) ≥ 100 U/L as poor prognostic factors [25]. A second report identifies the JCOG-Index; PS ≥ 1, number of metastatic sites ≥ 2, no prior gastrectomy, and ALP ≥ 100 U/L as risk factors [26]. External validation of these factors has been obtained from phase III clinical trials. Three reports from Korea [27–29] identified several prognostic factors, which are summarized in Table 2. There were three overlapping factors in these studies, namely PS, gastrectomy, and ALP levels.

**Table 2:** Prognostic factors in advanced gastric cancer

	Chau et al. [25]	Takahari et al. [26]	Lee et al. [27]	Kim et al. [28]	Koo et al. [29]	Fanotto et al. [31]	Fuchs et al. [41]
Host status							
ECOG PS	●	●	●	●	●	●	●
Tumor status							
No gastrectomy		●	●		●		●
Peritoneal metastasis	●		●	●	●		●
Bone metastasis			●	●	●		
Liver metastasis	●						
Lung metastasis					●		
Number of metastatic sites		●		●			
First-line TTP/PFS						●	●
Laboratory test values							
Increased ALP	●	●	●		●		●
Increased AST		●					●
Decreased albumin			●		●		●
Elevated total bilirubin				●	●		
Increased LDH						●	●
NLR						●	

ECOG PS: Eastern Cooperative Oncology Group performance status; TTP: time to progression; PFS: progression-free survival; ALP: alkaline phosphatase; AST: aspartate transaminase; LDH: lactate dehydrogenase; NLR: neutrophil-to-lymphocyte ratio. References [30] and [40] are reports on second-line treatment

In another report, independent prognostic factors for gastric cancer patients who underwent first-line treatment with S-1 plus cisplatin were PS ≥ 1, more than one metastatic site, and high ALP levels [30]. A report from Italy identifies PS, lactate dehydrogenase (LDH) levels, neutrophil-to-lymphocyte ratio (NLR), and PFS during second-line therapy as prognostic factors [31].

In advanced gastric cancer patients who underwent chemotherapy, high ALP levels were identified as an independent prognostic factor in multivariate analysis [32]. In a systematic review and meta-analysis of 76 clinical trials of solid cancers, high LDH levels were associated with poor prognosis [33]. In advanced gastric cancer, the albumin-globulin ratio (AGR: albumin/total protein—albumin) is an independent prognostic factor for OS and PFS [34]. In addition, a post hoc analysis of patients in the REAL-2 trial has reported on the prognostic value of the NLR [35].

In addition, LDH [36], VEGF [37], microsatellite instability (MSI) and HER2 [38], among others, have been indicated as biomarker candidates. Furthermore, there has been a report on nomograms combining HER2 status with other factors [39], as well as a report on another nomogram with seven factors [40] predicting survival.

With regard to second-line treatment, a pooled analysis of the RAINBOW and REGARD trials identified the following markers of poor prognosis: peritoneal metastases, PS 1, the presence of a primary tumor, time to progression (TTP) < 6 months, poor/unknown tumor differentiation, abnormally low blood levels of albumin, sodium, and/or lymphocytes, and abnormally high blood levels of neutrophils, aspartate aminotransferase (AST), ALP, and/or LDH [41]. Another study found PS 2, hemoglobin ≤ 11.5 g/dl, carcinoembryonic antigen (CEA) level > 50 ng/ml, the presence of three or more metastatic sites, and TTP under first-line chemotherapy of 6 months or less were poor prognostic factors [42]. Finally, PS 0–1, hemoglobin level ≥ 10 g/dl, and TTP under first-line therapy of 5 months or more were identified as favorable prognostic factors [43].

Although opinions on prognostic factors and the timing of treatment change vary, in one analysis conducted after the RAINBOW trial, it took 5.7 months to observe a reduction in PS by one grade in the ramucirumab group, but 4.3 months in the placebo group [44]. Similarly, in the TAGS trial, treatment with trifluridine/tipiracil prolonged the period to PS deterioration to > 2 compared to placebo [17]. In other words, transition to appropriate second- and subsequent lines of treatment may delay PS deterioration. On the other hand, the period from disease progression to PS deterioration is about 1 to 2 months. Therefore, if there is a decline in PS, it should be suspected that the disease may have already progressed.

If a patient has a factor related to poor prognosis, caution is warranted in regard to changes in their disease condition. If a deterioration in the parameter is observed, it is important to maintain the effectiveness of subsequent treatment by making an image-based diagnosis with PD in mind, and by monitoring the treatment effect on the whole body in a timely manner.

## Tumor markers

Tumor markers are very useful in some situations for monitoring the state of disease in clinical practice. However, an increase in tumor marker levels should not be the only reason for switching a patient onto a new chemotherapy regimen. The reports discussed below focus on detecting recurrent disease and distant metastasis after curative surgery, not on patients with advanced gastric cancer undergoing chemotherapy. For example, more than 90% of patients with elevated preoperative levels of CEA also have elevated CEA levels at the time of recurrence [45]. In addition, elevated levels of markers associated with tumor growth are observed 2–3 months before the emergence of imaging abnormalities. This means that only a small change may be detected in the radiological image when the levels of tumor markers increase during treatment for advanced gastric cancer. There is an obvious correlation between the increase in tumor burden and the elevation of tumor marker values. On the other hand, clinicians should abstain from easily changing the treatment regimen if there is no symptomatic exacerbation or radiographic progression. In other words, it is of utmost importance to utilize tumor markers properly and effectively when making the decision to change treatment.

There are some clinically useful tumor markers for monitoring gastric cancer, including CEA, carbohydrate antigen 19-9 in the sialyl Lewis A group (CA19-9), sialyl Tn antigen (STN), cancer antigen 72-4 (CA72-4), cancer antigen 125 (CA125), and alpha-fetoprotein (AFP) (Table 3) [46]. Each tumor marker has its own respective characteristics. For example, CEA has been significantly associated with differentiated tumor types [47], and CEA level is an independent predictive factor for the presence of liver metastasis. Although 5–10% of patients are Lewis negative, CA19-9 is frequently associated with nodal involvement; indeed, the positive predictive value of this marker for nodal involvement is 80% or more. On the other hand, the positive predictive value of CA19-9 for peritoneal metastasis is less than 30% [48]. High serum STN is an independent factor that predicts liver metastasis and a worse outcome in gastric cancer patients [49]. The rate of CA72-4 positivity is significantly higher than that of CEA in patients with poorly differentiated adenocarcinoma, in patients with type 4 gastric cancer, and in patients with peritoneal metastases; 36% vs. 8%, 67% vs. 11%, and 69% vs. 23%, respectively [50, 51]. The level of CA125 is significantly correlated with the degree of peritoneal dissemination and patient survival [52]. CEA and CA19-9 are the most frequently measured tumor markers for the monitoring of gastric cancer. However, patients are often negative for both CEA and CA19-9 upon initial presentation. Although the levels of these markers may increase in the late phase of the disease course, patients are often still classified as negative until the terminally ill stage. Therefore, clinicians must endeavor to find other markers of disease progression beyond CEA and CA19-9.

**Table 3:** Clinical relevance of serum tumor markers for gastric cancer

	T category	N category	M category	Peritoneal metastasis	Histology	Prognosis	Recurrence pattern
CEA	Yes	Yes	Yes	No	Yes	Yes	Distant
CA19-9	Yes	Yes	Yes	Yes	No	Yes	Distant
CA72-4	Yes	Yes	Yes	Yes	No	Yes	Distant and/or peritoneal
AFP	NA	NA	Yes	NA	Yes	Yes	Liver
CA125	NA	NA	NA	Yes	No	Yes	Peritoneal
STN	NA	NA	Yes	Yes	No	Yes	Peritoneal

NA: not enough evidence to evaluate clinical significance was available; CEA: carcinoembryonic antigen; CA19-9: carbohydrate antigen 19-9 in the sialyl Lewis A group; CA72-4: cancer antigen 72-4; AFP: alpha-fetoprotein; CA125: cancer antigen 125; STN: sialyl Tn antigen

In one example of utilizing tumor markers to inform the decision to change treatment, Hasegawa et al. [53] investigated patients with advanced gastric cancer with non-measurable peritoneal metastasis. These patients underwent treatment changes based on aggravated symptoms or elevated tumor markers, rather than on the radiological diagnosis of PD, and as a result had significantly improved OS. Peritoneal metastasis is often observed in diffuse-type adenocarcinoma, and there are many cases where no increase in tumor markers is observed in diffuse-type gastric cancer. However, Hasegawa et al. point out the utility of tumor markers with regard to making a decision to change treatment. Together, the findings of Hasegawa et al. suggest that tumor burden may already be high in patients whose treatment regimens are changed after PD is confirmed through CT scan results. As a cautionary note, that study was retrospective, and therefore, some bias may be involved; however, the overall importance of monitoring tumor markers is clearly demonstrated by that analysis.

In some exceptional cases, an initial elevation of tumor marker levels after initiation of chemotherapy doesn’t represent a sign of disease progression. This phenomenon is called a surge, which means a transient elevation of tumor marker levels despite achieving clinical benefit from chemotherapy. It has been reported that CEA and CA19-9 surges are observed in about 20% of patients, and the median time to peak is approximately 0.5–2 months after the initiation of chemotherapy [54].

## Radiographic assessments

Radiographic assessment is the key method for disease evaluation, and in this section we discuss the importance of computed tomography (CT) assessment and the appropriate timing of follow-up assessments. CT assessment is needed not only to analyze tumor response but also to evaluate disease progression. In clinical practice, physicians use various procedures to monitor a patient’s disease status. These include evaluation of patients’ symptoms, laboratory data (including tumor markers) and radiographic assessment with CT or other modalities. In order to assess signs or symptoms of disease progression, clinicians must routinely conduct radiographic assessments.

In most clinical trials of first-line chemotherapy, tumor evaluation is conducted every 6 or 8 weeks [2, 3, 7, 8]. Furthermore, in most clinical trials of second- or later-line chemotherapy, CT evaluation intervals are set at 6 weeks [11, 13, 14, 55]. Since PFS during second- or third-line chemotherapy is estimated to be shorter than PFS during first-line treatment, we suggest that detailed evaluation should be carried out more often, which could be achieved through shortening the interval between CT scans.

If patients have only non-measurable lesions such as peritoneal or bone metastases with or without a primary gastric tumor, clinicians must pay close attention to signs of disease progression. It is difficult to detect signs of disease progression only from physical findings or symptoms, or laboratory data, including tumor markers. Therefore, by performing radiographic assessments routinely, clinicians will be much less likely to miss disease progression, and will detect it before clinical symptoms appear. When there is a suspicion of disease progression, even if signs of disease progression are not detected through the most recent CT evaluation, clinicians should consider the use of another radiographic assessment modality such as barium enema, or ultrasonography, to determine whether peritoneal metastasis is present. For the detection of peritoneal metastasis, CT showed a higher sensitivity (76.5%) compared with ^18^F-FDG PET (35.3%), although CT had a relatively lower specificity (91.6%) than did PET (98.9%) [56]. If patients have a primary gastric tumor, an upper gastrointestinal endoscopy is recommended to evaluate the disease condition directly.

## Determining disease progression by symptoms

In contrast to other carcinomas, non-target lesions (including peritoneal dissemination) are common in gastric cancer. The frequency of peritoneal metastasis reported in the G-SOX [8], SPIRITS [3], and START [57] trials of first-line chemotherapy was 20%, 29% and 39%, respectively, while that reported in the WJOG4007 [11], REGARD [13], KOREA [12], and RAINBOW [14] trials of second-line chemotherapy was 26%, 31%, 45% and 47%, respectively. Since patients with severe peritoneal dissemination are excluded from such trials, its actual frequency is higher in clinical practice. In addition, because peritoneal metastasis is accompanied by various clinical symptoms that preclude an objective evaluation of disease status, the opportunity for further treatment can be missed due to rapid disease progression.

In clinical trials, tumor response is evaluated according to the revised RECIST guidelines (version 1.1). The original RECIST v1.0 clearly stated that “it is not intended that these guidelines will be used as a basis for making decisions about continued therapy”; RECIST v1.1 also takes the same position, stating that “Many oncologists in their daily clinical practice follow their patients’ malignant disease by means of repeated imaging studies and make decisions about continued therapy on the basis of both objective and symptomatic criteria. It is not intended that these RECIST guidelines play a role in that decision-making, except if determined appropriate by the treating oncologist.”

Consequently, “overall response” based on the results of response evaluation according to the RECIST guidelines “should be used to determine whether an agent or regimen shows a promising result that is worth continuing development research.” In other words, determining whether to continue the therapy or not for an individual patient should not be based on overall response (complete response/PR/SD/PD) but on comprehensive “clinical decisions” including imaging results, symptoms, physical findings, and different examinations. Accordingly, the continuation of protocol treatment may still be clinically appropriate in some patients, even when PD is determined as the overall response based on diagnostic imaging. In these cases, determining whether to continue protocol treatment should be based on clinical decisions regardless of overall response. Reciprocally, protocol treatment should be discontinued in cases that are not diagnosed as PD by response evaluation criteria based on imaging results, but are determined clinically and comprehensively by physicians to be a ‘clinical exacerbation’.

The original RECIST v1.1 publication states in special notes on assessment of progression of non-target disease that “to achieve ‘unequivocal progression’ on the basis of the non-target disease, there must be an overall level of substantial worsening in non-target disease such that, even in the presence of SD or PR in target disease, the overall tumor burden has increased sufficiently to merit discontinuation of therapy”. This means that the evaluation of PD in non-target lesions affects “decisions on whether to continue a therapy or not for each patient” and in consequence generates confusion. It should be noted that such “unequivocal progression” is an evaluation criterion limited solely to the evaluation of “PD in non-target lesions.”

Peritoneal dissemination may cause serious complications, such as intestinal obstruction, massive ascites and hydronephrosis associated with the clinical presentation of abdominal pain and feeling of fullness, vomiting, constipation, malnutrition and renal dysfunction (Table 4) [58]. The overall response rate has not been adopted as an endpoint in clinical studies because a substantial proportion of gastric cancer patients with peritoneal metastasis do not have measurable lesions according to the RECIST criteria, and clinical symptoms are more important in determining whether to discontinue treatment. Moreover, it is often difficult to differentiate treatment toxicity from disease progression in patients with complicating severe anorexia and/or nausea. In these cases, the decision to discontinue treatment (a Go/No-Go decision) should be made after a relatively short follow-up period.

**Table 4:** Findings of disease progression involving peritoneal metastasis

Progression of peritoneal metastasis
Aggravation of peritoneal mass, intestinal wall thickening, ascites, and intestinal stenosis
Aggravation of intestinal obstruction or stenosis symptoms, such as decreased food intake, abdominal pain, nausea and vomiting not attributable to chemotherapy-related adverse events
Aggravation of the feeling of abdominal fullness
Deterioration of renal function (emergence of hydronephrosis due to aggravation of peritoneal dissemination)
Deterioration of general condition such as a decrease in performance status determined to be caused by the original disease
An increase in the number of times that ascites drainage is required
Disease progression difficult to determine from imaging test results
Exacerbation of cancer pain
Deterioration of general condition such as a decrease in performance status determined to be caused by the original disease
The emergence and worsening of disseminated intravascular coagulation
An increase in tumor markers

We analyzed salvage therapy trials where the active compound was compared to placebo plus best supportive care. In that type of trial, the high frequency of symptoms seen in the placebo arms as adverse events could easily be expected to suggest signs of disease progression (Table 5) [15, 17].

**Table 5:** Adverse events in the placebo arms of phase 3 trials in the salvage-line setting

	REGARD (*n* = 115)		TAGS (*n* = 168)	
	Any grade (%)	Grade ≥ 3 (%)	Any grade (%)	Grade ≥ 3 (%)
Anorexia	23	3	30	6
Nausea	–	–	32	3
Vomiting	25	4	20	2
Dysphagia	10	4	4	2
Abdominal pain	28	3	19	9
Diarrhea	–	–	15	2
Constipation	23	3	12	2
Fatigue	40	10	21	6
Dyspnea	13	6	10	3

Therefore, if clinical symptoms or abnormal blood test data (including renal dysfunction and elevated bilirubin, or elevated tumor markers, ALP, and LDH) suggest there is an exacerbation of the disease, imaging tests should be promptly carried out. In addition to imaging test results, the clinical symptoms and blood test data must be taken into consideration and, if determined necessary, transition to a subsequent treatment should be considered.

## Summary

**Fig. 2:**
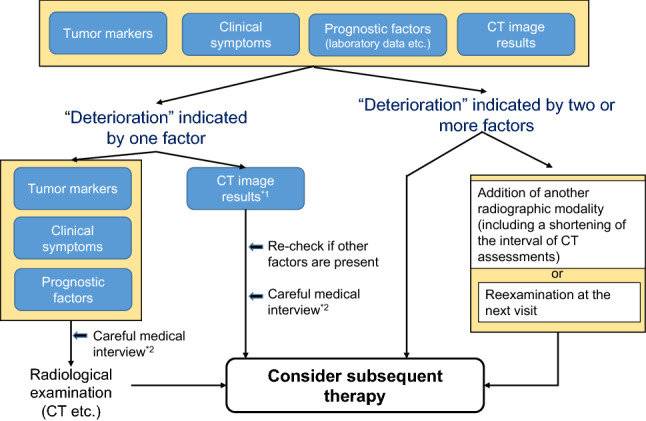
The decision to change treatment in patients with advanced gastric cancer receiving chemotherapy. *1 Deterioration indicated by CT image results includes the following; an increase in the size of the target lesion; an increase/trend for an increase in the size of non-target lesion; emergence of a new lesion; characteristic findings (bowel wall thickening, bowel dilatation, hydronephrosis, biliary dilatation, etc.). *2 A careful medical interview includes questions about; the condition of meal intake; body weight changes; symptoms such as constipation/diarrhea, feeling of fullness, relapse of previous symptoms(not only abdominal pain and nausea); other changes in daily life

Clinicians need to judge treatment decision based on the evaluation of tumor markers, prognostic factors, radiographic assessment and clinical symptoms (Fig. 2).

1. The status of tumor markers, such as CEA, CA19-9 and other markers indicative of specific sites of metastasis, should be taken into account when determining whether to change treatment in advanced gastric cancer. Clinicians should also be aware of the possibility of a transient surge in tumor marker levels just after initiation of chemotherapy despite the patient achieving clinical benefit from it.
2. When patients have many poor prognostic factors, such as poor PS, increased ALP levels, and peritoneal metastasis, they should be monitored more closely to avoid missing the opportunity for a timely switch in treatment regimens.
3. Though RECIST criteria are important in terms of response evaluation, clinical decisions should not be based solely on radiologic findings but should take into consideration other findings.
4. Clinicians should take into consideration symptoms, such as bowel fullness and nausea indicative of peritoneal metastasis, and specific changes in blood examination results and imaging test results. If such findings have changed, imaging tests should be carried out promptly to make a decision for Go/No-Go of the current regimen.

## Conclusion

Gastric cancer tends to progress rapidly in a short period, and without careful patient follow-up, physicians may miss the appropriate opportunity for switching to a subsequent therapy. Unlike 10 years ago, many drugs that can significantly extend survival time are now available and patients should never be deprived of the opportunity to access such effective drugs. It is of utmost importance to comprehensively capture signs that indicate exacerbation of the disease. In clinical practice, physicians sometimes face paradoxical situations such as an increase in a clinical parameter of disease worsening despite CT imaging results showing tumor shrinkage. Halting disease progression in patients is a paramount concern for clinicians. Therefore, it is critical that multiple factors are taken into account when deciding on the best clinical course of action. Needless to say, with this added level of extra care, significant improvements can be expected in the prognosis of patients with gastric cancer.
